# Spectrum of spinal cord space occupying lesions: An observational perspective from Pakistan

**DOI:** 10.12669/pjms.41.13(PINS-NNOS).13440

**Published:** 2025-12

**Authors:** Zia-Ul-Rehman Najeeb, Naeem-Ul-Hassan, Inaam Elahi, Muhammad Jehanzeb, Ali Azan Ahmed, Haseeb Mehmood Qadri

**Affiliations:** 1 Zia-Ul-Rehman Najeeb, MBBS. Punjab Institute of Neurosciences, Lahore, Pakistan; 2 Naeem-Ul-Hassan, MBBS, MS. Punjab Institute of Neurosciences, Lahore, Pakistan; 3 Inaam Elahi, MBBS, FCPS. Punjab Institute of Neurosciences, Lahore, Pakistan; 4 Muhammad Jehanzeb, MBBS, MS. Punjab Institute of Neurosciences, Lahore, Pakistan; 5 Ali Azan Ahmed Medical Student, Aga Khan University, Medical College, Karachi, Pakistan; 6 Haseeb Mehmood Qadri, MBBS. Punjab Institute of Neurosciences, Lahore, Pakistan

**Keywords:** Pakistan, Spinal cord tumors, Spinal meningomas, Spinal astrocytomas, Spinal metastasis

## Abstract

**Objective:**

To analyze the clinico-radiological characteristics and neurosurgical management of spinal space-occupying lesions (SOLs).

**Methodology:**

A retrospective observational study involving patients with spinal cord SOLs who underwent surgical management at the Department of Neurosurgery, Unit-I, Punjab Institute of Neurosciences, between 2022 and 2024, was conducted after approval from the institute’s review board. Patient profiles, clinical and radiological features, tumor location, and surgical outcomes were analyzed for comparative assessment.

**Results:**

With a mean age of 36.78 ± 16.57 years, a clear male preponderance of 62.5% (35) was noted for spinal SOLs in a Pakistani cohort of 56 patients. Among all spinal SOLs, 46.4% (26) were intradural intramedullary (IDIM) lesions, 25% (14) were intradural extramedullary (IDEM), and 28.6% (16) were extradural (ED) lesions. The most common symptoms were lower back pain in 65.4% (17) in IDIM, 68.8% (11) in ED, and 64.3% (9) in IDEM lesions. Post-operatively, Karnofsky Performance Scale (KPS) score was improved in all patients. The most common histopathology was meningioma in 76.9% (20) of IDIM patients, astrocytoma in 71.4%(10) of IDEM patients, and hemangioma in 62.5% (10) of ED patients.

**Conclusion:**

Patients with spinal lesions significantly improved clinically post-operatively, regardless of the anatomical location of the lesion. Surgical intervention remained the primary treatment for majority patients with spinal cord SOLs..

## INTRODUCTION

Spinal space-occupying lesions (SOLs) are a fascinating group of lesions that account for a small proportion among central nervous system (CNS) lesions but are often capable of causing significant morbidity. Among CNS tumors, only 15% occur intraspinal.^1^ Most of the intraspinal SOLs are benign in nature. The estimated incidence of spinal tumors in the general population ranges from 0.74 to 1.6 cases per 100,000 persons per year.^2^ Depending on their location in the spinal cord, these lesions are further broadly classified into extradural (ED) and intradural (ID) spinal lesions. Among these, 60% of tumors are ED, while 30% account for intradural extramedullary (IDEM) and only 10% SOLs are intradural intramedullary (IDIM) spinal cord tumors.^1^ Among IDEM tumors, the two most common tumors are meningioma and schwanomma, meningioma being more common (30.7%) and schwanomma second most common accounting for 15.5.%. Both of these are benign tumors, mostly WHO grade 1. Although meningiomas are mostly Grade-1 tumors with female predominance but among male patients, they can be more aggressive, showing WHO Grade-2 and 3 on histopathology, showing more aggressive behavior. Thus, it requires a more aggressive management approach.^3^ Very rarely, IDEM tumors are malignant with a poor prognosis.^4^ Presenting symptoms in adults are usually recumbent pain, but more specifically, symptoms arise when EM tumors start compressing the spinal cord, thus making neurological symptoms dictated by the location of SOLs. As per the benign nature of these tumors, symptoms appear late due to slow growth and delayed compression, thus requiring early investigations for the timely initiation of treatment to avoid debilitating neurological deficits.^1^ IDIM SOLs are a rare entity, accounting for only 10% of spinal tumors. Ependymomas and low-grade astrocytomas are the most common histopathological types seen in this category.^5^,^6^ Ependymomas are mostly benign, comprising 15% of intramedullary tumors, mostly occurring in the adult population, whereas astrocytomas account for 30%, mostly occurring in the pediatric population in association with neurofibromatosis 1 (NF1) and are more infiltrative, thus making gross resection difficult to achieve.^5^ Other rare entity in this category include dermoid cyst.^7^ Among IM tumors, histopathological type, grade, and location of the tumor play major roles as predictive factors. Dorsal localization of tumors and tumors of the cervico-occipital junction show poor prognosis and poor functional outcomes. Similarly, ependymoma shows better functional outcome after surgical resection as compared to astrocytoma because its infiltrative nature does not allow complete excision.^3^ Generally, because of the benign and non-infiltrative nature of spinal tumors, they are managed easily by gross total resection (GTR) of tumors, rarely requiring any adjuvant therapy except in cases of astrocytoma, specifically high grade, in which infiltrative nature of tumor allows GTR only in selected cases and adjuvant therapy after debulking as main stay of management.^1^

Spinal tumors, despite being small in numbers among CNS tumors, are important entities due to their effect on the major neurological system of the body, which can make a patient handicapped to a debilitating extent if not diagnosed and managed timely. Despite their importance, we do not have PubMed-indexed data available considering Pakistani prevalence, which can help us devise proper management plans. We planned to review the data of patients presenting to our tertiary care center with spinal tumors.

## METHODOLOGY

A retrospective-prospective observational study was conducted in January-March 2025 at the Department of Neurosurgery, Unit-I, Punjab Institute of Neurosciences, after due approval from the Institutional Review Board (IRB). The study included records from the last three years, i.e. January 01, 2022 to December 31, 2024. Neurosurgeons with at least ten years of clinical experience performed surgeries upon the included patients during the mentioned duration. The sampling technique employed was non-probability consecutive sampling.

### Ethical declaration:

This study was approved by the institutional review board on February 19, 2024 with approval number 1727/IRB/PINS/Approval/2024.

### Inclusion Criteria

- Operated on cases of primary spinal cord SOL
- Age 18 years or more, inclusive of all genders

### Exclusion criteria

- Patient with a diagnosis of secondary spinal cord SOL
- Age less than 18 years
- Non-operative cases
- Incomplete follow-up and incomplete record

### Data analysis technique:

Data was collected by accessing the picture archiving and communication system (PACS) and patient records. Demographic details, signs, symptoms, duration of disease, examination findings, co-morbidities, radiological findings, tumor characteristics, surgical approach, and post-operative sequelae were recorded. Furthermore, each patient was assigned a unique study ID against their medical registration (MR) number, and Google Forms (Google Inc., USA) was used as a data collection tool. Data de-identification was ensured by using the patient ID while collecting data. The data was analyzed using Statistical Package for the Social Sciences (SPSS) version 26.0. Descriptive analysis was done to compute the results, along with measures of central tendency and percentage.

## RESULTS

In our study, 56 patients were diagnosed with a spinal lesion, 62.5% (35) were male, and 37.5% (21) were female, with an overall mean age of 36.78 ± 16.57 years. Among all spinal SOLs, 46.4% (26) were IDIM lesions, 25%(14) were IDEM, and 28.6% (16) were ED lesions This study comprises 46.4% (12) males and 53.8% (14) with IDIM SOLs, and 78.6% (11) males and 21.4% (3) with IDEM SOLs, and 75% (16) males and 25% (4) females having ED SOL.

In patients with IDIM, the most common clinical presentation was lower back pain in 65.4%(17), radicular pain, and motor deficit in 73.1% (19) with bilateral paraparesis in 3.85% (1) and in patients with IDEM SOL most common presentation was lower back pain in 64.3% (9) along with radicular pain and motor deficit was 50%(7) and 57.1%(6), respectively along with bilateral paraparesis in 14.3% (20). In addition, patients with ED SOL mostly presented with lower back pain in 68.8% (11), with radicular pain and motor deficit in 75% (12) and 56.3% (9), respectively, associated with bilateral paraparesis in 12.5% (2). However, in all patients, GCS was in the range of 13-15. The preoperative mean Karnofsky Performance Scale (KPS) score in patients with IDIM SOL was 60 ± 12, 58.57 ± 16.4 in IDEM, and 65 ± 11.5 in ED SOLs (Table-I).

**Table-I:** Descriptive analysis of the demographics and clinical presentation of patients with spinal SOLs.

Variables	IDIM	IDEM	ED
Patient distribution, % (n)	26 (46.4%)	14 (25.0%)	16 (28.6%)
Gender distribution, M/F, % (n)	12 (46.2%) Males 14 (53.8%) Females	11 (78.6%) Males 3 (21.4%) Females	12 (75.0%) Males 4 (25.0%) Females
Symptoms at Presentation, % (n)	Lower back pain - 17 (65.4%) Local or radicular pain - 19 (73.1%) Motor deficits - 19 (73.1%) Sensory symptoms - 8 (30.8%) Sphincter disturbances - 3 (11.5%) Neck pain - 3 (11.5%) Nausea - 1 (3.85%)	Lower back pain - 9 (64.3%) Local or radicular pain - 7 (50.0%) Motor deficits - 8 (57.1%) Sphincter disturbances - 2 (14.3%) Neck Pain - 2 (14.3%) Sensory symptoms - 1 (7.14%) Nausea - 1 (7.14%)	Lower back pain - 11 (68.8%) Local or radicular pain - 12 (75.0%) Motor deficits - 9 (56.3%) Sensory symptoms - 8 (50.0%) Neck pain - 5 (31.3%) Sphincter disturbances - 1 (6.25%) Progressive lower limb paresis - 1 (6.25%)
***Pre-operative GCS, % (n)***			
3-8	0	0	0
9-12	0	0	0
13-15	26 (100%)	14 (100%)	16 (100%)
Pre-operative Neurology, % (n)	Paraparesis - 1 (3.85%)	Paraplegia/Paresis - 2 (14.3%)	Paraparesis/Paraplegia - 2 (12.5%)
		Sensory loss in dermatomes - 1 (7.14%)	
		Saddle anesthesia - 1 (7.14%)	
		General malaise - 1 (7.14%)	
Pre-operative KPS, mean ± SD	60±12	58.57±14.6	65±11.55

On radiology, non-enhanced CT Spine in patients with IDIM showed a hyperintense lesion in 46.1% (12), a hypointense lesion in 53.8% (14). In patients with INEM, all 100% (14) of patients have a hypointense lesion on non-enhanced CT spine. Whereas, in patients with ED lesions, 68% (11) of the lesions were hypointense, 25% (4) of the lesions were hyperintense, and 6.25% (1) were calcified. Furthermore, MRI revealed that 96.2% (25) of lesions with IDIM were hypointense on T1-weighted imaging, while hyperintensity on T2-weighted sequences was observed in all 100% (26) of patients. On enhanced MRI, 76.9% (20) lesions were homogenously enhanced and 23.1% (6) were heterogenously enhanced. In patients with IDEM lesions, all 100% (15) lesions were hypointense, 92.9% (14) of lesions were hyperintense, and 92.9% (13) of lesions were homogenously enhanced on enhanced MRI. In patients with ED lesions, 68% (11) of lesions were hypointense, 31.3% (5) lesions were hyperintense on T1-weighted imaging, 87.5% (14) of lesions were hyperintense, 12.5% (2) of lesions were hypointense on T2-weighted imaging and 62.5% of lesions showed multiple flow voids in enhanced MRI, heterogenous enhancement in 31.25% (5) of lesions and homogenous enhancement in 6.25% (1) of lesions.

All patients underwent excisional surgery, and none of the patients needed CSF diversion surgery. Regarding IDIM, lesions were most common in the dorsal region in 42.3% (11), IDEM lesions were most common in the dorso-lumbar region in 28.6% (4) of patients, followed by dorsal in 21.4% (3) of patients, and ED lesions were common in 37.5% (6) and cervical region in 31.3% (5).

Post-operatively, in all patients, GCS was between 13-15, and KPS with standard deviation was 68.07±16.81 in patients with IDIM lesions, 59.29±17.30 in patients with IDEM lesions, and 73.32±13.02 in patients with ED lesions. On histopathology, in patients with IDIM, the most frequent lesions were meningioma in 76.9% (20) of patients, followed by schwannoma in 23.1% (6) of patients. In patients with IDEM, 71.4% (10) had astrocytomas, followed by ependymoma in 28.5% (4) of patients. Additionally, in patients with ED lesions, 62.5% (10) were hemangioma, 18.8% were osteoma, and 12.5% (2) were metastasis (Table-II).

**Table-II:** Descriptive analysis of parameters of surgical management, radiological findings, and post-operative sequelae of patients with spinal SOLs.

Variables	IDIM	IDEM	ED
Level of involved Spinal Cord, % (n)	Cervical - 5 (19.2%)	Cervical - 2 (14.3%)	Cervical - 5 (31.3%)
	Cervico-dorsal - 1 (3.8%)	Cervico-dorsal - 1 (7.1%)	Dorsal - 6 (37.5%)
	Dorsal - 11 (42.3%)	Dorsal - 3 (21.4%)	Lumbar - 2 (12.5%)
	Lumbar - 3 (11.5%)	Lumbar - 1 (7.1%)	Dorso-lumbar - 2 (12.5%)
	Lumbo-sacral - 1 (3.8%)	Lumbo-sacral - 2 (14.3%)	Sacral - 1 (6.3%)
	Dorso-lumbar - 5 (19.2%)	Dorso-lumbar - 4 (28.6%)	
		Dorso-lumbo-sacral - 1 (7.1%)	
Side of SOL, % (n)	All midline	All midline	All midline
MRI T1	Hypointense: 25 (96.2% )	Hypointense: 15 (100% )	Hypointense: 11 (68%) Hyperintense: 5(31.3% )
MRI T2	Hyperintense: 26 (100% )	Hyperintense: 14 (92.9% )	Hyperintense: 14 (87.5% ) Hypointense: 2 (12.5% )
Enhanced MRI	Homogenous: 20 (76.9% ) Heterogenous: 6 (23.1%)	Homogenous: 13 (92.9% )	Multiple flow voids: 10 (62.5% ) Heterogenous: 5 (31.25% ) Homogenous: 1 (6.25%)
***Consistency, % (n)***			
Soft	4 (15.4%)	3 (21.4%)	5 (31.3%)
Firm	14 (53.8%)	9 (64.3%)	8 (50.0%)
Hard	8 (30.8%)	2 (14.3%)	3 (18.8%)
***Vascularity, % (n)***			
Mild	3 (11.53%)	3 (21.4%)	1 (6.25%)
Moderate	7 (26.9%)	2 (14.3%)	7 (43.8%)
High	16 (61.5%)	9 (64.3%)	8 (50.0%)
***Extent of Resection, % (n)***			
GTR	19 (73.1%)	0	12 (75.0%)
NTR	7 (26.9%)	2 (14.3%)	4 (25.0%)
STR	0	12 (85.7%)	0
PR	0	0	0
Biopsy	0	0	0
***Post-operative Complications, % (n)***			
New Neurologic Deficit			
Surgical Site Infection	0	0	0
CSF Leak	0	0	0
Hydrocephalus	0	0	0
Others	0	0	0
	Lower back pain, 13 (50.0%), Neck pain, 2 (7.69%)	Lower back pain, 5 (35.7%), Neck pain, 1 (7.14%)	Lower back pain, 8 (50.0%), Neck pain, 3 (18.8%), Paresthesia in lower limbs, 1 (6.25%)
Post-operative KPS, mean ± SD	68.07±16.81	59.29±17.30	73.32±13.02
Histopathology, % (n)	Meningioma, 20 (76.9%) Schwannoma, 6 (23.1%)	Astrocytoma, 10 (71.4%) Ependymoma, 4 (28.5%)	Hemangiomas, 10 (62.5%) Osteoid Osteoma, 3 (18.8%) Metastasis, 2 (12.5%) Chordoma, 1 (6.25%)

## DISCUSSION

Despite the global prevalence of spinal SOLs, with 15% of all CNS tumors occurring intraspinally, there is limited information of SOLs on the clinicopathologic profile of these patients. Moreover, the anatomic location of spinal SOLs is critical to both diagnosis and treatment, and current literature provides limited insight into correlations between anatomic location, patient presentation, and outcomes. These gaps in understanding the clinical presentation, diagnostic mechanisms, histopathological profile, and treatment create significant lapses in patients’ prognosis and treatment outcomes. Our retrospective observational study aimed to explore the clinicopathological profile of patients, establishing a set of parameters that inform improved surgical management and patient outcomes.

Among the 56 patients, 35 were males and 21 were females. IDIM 46.4% (26) was the most common tumor site, followed by ED 28.6% (16) and IDEM 25.0% (14). IDIMs had a nearly equal sex distribution between males, 46.2% (12), and females, 53.8% (14). However, IDEMs were more common in males, 78.6% (11), than in females, 21.4% (3). Similarly, EDs were also more prevalent in males, 75.0% (12), than in females, 25.0% (4).

According to studies, IDEM is the most commonly occurring spinal SOL, followed by ED and IDIM. Jignesh et al.’s study of 178 patients in India reported IDEMs 65.73% (117) to be the most common tumor.^8^ Similarly, Dikondwar et al.’s study of 72 patients across five years reported IDEM 40% (30) as the most prevalent SOL compartment.^9^ However, our findings report that IDIMs had the highest prevalence at 46.4% (26), followed by ED 28.6% (16) and IDEM 26.0% (14).

In terms of clinical presentation, Jignesh et al. report motor deficit 75.3 % (134) as the primary complaint, followed by paresthesia 46.6 % (83) and sensory loss 41.5 % (74).^8^ Similarly, Dikondwar et al. reported nerve root pain 57.9% (41), back pain 54% (39), and paresthesia 43% (31) as the chief complaints.^9^ Hachiha et al. ’s retrospective study of 120 patients also showed motor trouble as the primary complaint in 77.5% (93) of patients, followed by spinal pain in 39.2% (47) and radicular pain in 24.2% (29).^5^ Jecko et al. reported motor deficit in 52% of patients presenting with IDIM.^10^ Constantini et al. also reported motor disturbance as the most frequent complaint at the time of diagnosis, with 61.8% (62) of patients, followed by sensory deficits in 42.2% (42).^11^

However, our findings report that the chief complaints vary across the different types of SOLs. While motor deficits 73.1% (19) were the chief complaint in IDIM patients, lower back pain 64.3% (9) was more frequent in IDEM patients, and local or radicular pain 75.0 % (12) was more frequent in ED patients. In addition, our findings also report cases with dedicated complaints such as sphincter disturbances, neck pain, nausea, and sensory symptoms. While the overall set of presenting complaints in spinal SOL patients remains the same, our findings conclude that the frequency of chief complaints varies according to each compartment.

Radiologically, our findings report that IDEM cases presented with all hypointense lesions 100%, compared to one hyperintense lesion in IDIMs (3.85%) and five hyperintense ED lesions, 31.3% (5) on the T1-weighted images (T1WI). Moreover, MRI T2 scans showed all IDIM (100%) cases as hyperintense compared to IDEMs (92.9%) and EDs (87.5%). Our radiological findings are consistent with the current literature. Parsa et al. and Grimm et al. both report all IDIMs as hypointense on T1W MRI scans and hyperintense on T2 MRI scans.^12^,^13^

Among all tumor categories in our cohort, the dorsal spine was the most commonly affected spinal segment. Dorsal lesions were found in 42.3% (11) of individuals with IDIM malignancies. Similarly, IDEM tumors mainly affected the dorso-lumbar junction, 28.6% (4), but extradural (ED) tumors were more likely to involve the dorsal region, 37.5% (6). With literature reporting the thoracic segment as the most commonly affected region, 80% of cases by Ottenhausen et al.^1^, 33% of cases by Dikondwar et al.^9^ and 37.64% by Jignesh et al.^8^ Our findings are also reinforced by Aftab et al.’s study of 52 patients reporting dorsal lesions, the most common at 51.9% of cases.^14^

All of our 56 patients, IDIMs, IDEMs, and EDs, underwent excisional surgery for treatment. GTR was performed for 19 cases of IDIM (73.1%) and 12 cases of ED (75.0%). Subtotal resection was preferred for IDEM cases, with 85.7% of cases, while some patients underwent near-total resection.

Our study reported limited post-op complications, with lower back pain being the most common in 50% of IDIM cases, 35.7% IDEM cases, and 50% of ED cases. No complaints of hydrocephalus, new neurologic deficit, surgical site infection, or CSF leak were recorded. Our findings show reduced complications with GTR compared to study by Jignesh et al. where 26 patients had persistent post-op pain, nine patients developed neurologic deficits, and nine patients had CSF leak.^8^ Hachiha et al. had similar success with GTR and STR, resulting in favourable outcomes in 84.2% of patients with few infectious and embolic complications.^5^

In our study, meningioma 76.9% (20) was the most common IDIM, astrocytoma 71.4% (10) in IDEMs, and hemangioma 62.5% (10) in ED patients. Literature reports that schwannomas are the most common IDEMs and EDs, while ependymomas are more frequent in IDIMs. Jignesh et al. report 63.2% IDEM schwanommas, 34.7% ED schwanommas, and 73.6% IDIM ependymomas.^8^

The KPS, which measures functional status, revealed significant variations between tumor types before and after surgery. The mean KPS of patients with IDIM lesions was 60 ± 12 before surgery, and it increased to 68.07 ± 16.81 after surgery. Likewise, patients with extradural (ED) tumors showed a notable functional recovery, improving from a pre-operative KPS of 65 ± 11.55 to a post-operative KPS of 73.32 ± 13.02. Patients who had IDEM tumors, on the other hand, had a lower pre-operative KPS of 58.57 ± 14.6 and only slightly improved to 59.29 ± 17.30 after surgery. The evaluation of 128 patients with spinal SOLs by Safavi-Abbasi et al. reported an increase in KPS post-operatively (p = 0.001).^15^ Similarly, retrospective study of 31 spinal SOL cases by Patel et al. reported an increase in KPS scores from 61.18 ± 6.97 (preoperatively) to 65.29 ± 9.43 (postoperatively) (p < 0.05).^16^ Our findings are consistent with current literature of a marked increase in KPS scores and also add dedicated KPS scores for spinal SOLs based on anatomic location.

### Limitations:

First, the relatively small sample size in each subgroup (IDIM, IDEM, and ED) limits the generalizability of the findings. Small subgroup sizes reduce the statistical power of our analyses, increasing the likelihood of type-II errors and limiting the ability to detect subtle differences or associations. Furthermore, the limited sample diversity may restrict the applicability of our results to broader patient populations or different clinical settings.

## CONCLUSION

These findings imply that, compared to IDEM cases, patients with ED and IDIM tumors benefited more from surgical treatment. The increased vascularity and intricate anatomical dissection planes linked to these injuries may be the cause of the IDEM group’s comparatively small recovery, which could result in more severe neurological impairments. Overall, stabilization or an improvement in functional status was linked to surgical management of spinal SOLs in all groups, highlighting the importance of prompt intervention.

### Future Directions:

To confirm these findings, larger multicenter investigations would be required. Second, the study’s design was retrospective, which raises the possibility of insufficient data collection and selection bias, especially concerning long-term consequences and problems. Lastly, there was no thorough assessment of long-term neurological healing, recurrence rates, or quality of life in the post-operative follow-up data, which was mainly concerned with immediate functional outcomes like KPS scores.

### Author`s Contribution:

**ZURN, AAA:** Literature search, Data interpretation, critically reviewed manuscript.

**NUH, AS:** Concept and design of the work, Critical review of the manuscript.

**IE, HMQ:** Data analysis and interpretation, Critically reviewed manuscript and Supervision.

All authors have approved the final version to be published. They are also accountable for the integrity of the study.
